# Effects of *OsCDPK1* on the Structure and Physicochemical Properties of Starch in Developing Rice Seeds

**DOI:** 10.3390/ijms19103247

**Published:** 2018-10-19

**Authors:** Jian-Zhi Jiang, Chun-Hsiang Kuo, Bo-Hong Chen, Mao-Kei Chen, Choun-Sea Lin, Shin-Lon Ho

**Affiliations:** 1 Department of Agronomy, National Chiayi University, Chiayi 60004, Taiwan; ssdog27@yahoo.com.tw (J.-Z.J.); efab888@yahoo.com.tw (C.-H.K.); chen.po.hung9117@gmail.com (B.-H.C.); fritz29522879@yahoo.com.tw (M.-K.C.); 2 Agricultural Biotechnology Research Center, Academia Sinica, Taipei 11529, Taiwan; cslin99@gate.sinica.edu.tw

**Keywords:** rice, *OsCDPK1*, seed development, starch biosynthesis, endosperm appearance

## Abstract

Overexpression of a constitutively active truncated form of *OsCDPK1* (*OEtr*) in rice produced smaller seeds, but a double-stranded RNA gene-silenced form of *OsCDPK1* (*Ri*) yielded larger seeds, suggesting that *OsCDPK1* plays a functional role in rice seed development. In the study presented here, we propose a model in which *OsCDPK1* plays key roles in negatively controlling the grain size, amylose content, and endosperm appearance, and also affects the physicochemical properties of the starch. The dehulled transgenic *OEtr* grains were smaller than the dehulled wild-type grains, and the *OEtr* endosperm was opaque and had a low amylose content and numerous small loosely packed polyhedral starch granules. However, the *OEtr* grain sizes and endosperm appearances were not affected by temperature, which ranged from low (22 °C) to high (31 °C) during the grain-filling phase. In contrast, the transgenic *Ri* grains were larger, had higher amylose content, and had more transparent endosperms filled with tightly packed polyhedral starch granules. This demonstrates that *OsCDPK1* plays a novel functional role in starch biosynthesis during seed development and affects the transparent appearance of the endosperm. These results improve our understanding of the molecular mechanisms through which the grain-filling process occurs in rice.

## 1. Introduction

The quality of rice (*Oryza sativa* L.) grain is defined in terms of several main factors, including (i) eating and cooking qualities, and (ii) milling qualities and appearance [1]. Eating and cooking qualities are determined by the amylose content, amylopectin structure, gelatinization temperature, and pasting viscosity [2], and milling qualities and appearance correlate strongly with the transparency, flouriness, and chalkiness of the endosperm [3]. The filling and accumulation of starch granules in developing rice endosperms can accelerate at high temperatures, causing the starch in the endosperm cells to be packed loosely and the kernel to be chalky. Such grains crack easily during milling, yielding poor eating and cooking qualities [4,5,6].

It has been shown in many studies that chalky and less-transparent kernels contain more amylopectin and less amylose in the endosperm than do less-chalky and more-transparent kernels [7,8,9]. Eliminating chalkiness by regulating the amylopectin and amylose content ratios (by affecting biosynthesis) in the endosperm during the grain-filling phase is therefore a key way of improving grain quality. Two enzymes involved in amylose biosynthesis are ADP-glucose pyrophosphorylase and the Waxy gene-encoded granule-bound starch synthase I (GBSSI) [10,11]. The enzymes involved in the biosynthesis and modification of amylopectin are ADP-glucose pyrophosphorylase, soluble starch synthase, the starch-branching enzyme (BE), and the starch-debranching enzyme [10,11,12]. In higher plants, BE plays an essential role in amylopectin biosynthesis because it is the only enzyme that can add α-1,6-glucosidic linkages to polyglucans [13]. Three BE isoforms—BEI, BEIIa, and BEIIb—have been found in rice [10,14]. The rice mutant *amylose-extender*, which has a null mutation in *BEIIb*, has been found to alter the degree of polymerization (DP) of amylopectin, giving fewer short chains (DP ≤ 17) and more long chains (DP ≥ 18), the changes being related to the dose on the *amylose-extender* locus in the triploid endosperm cells. These results suggest that *BEIIb* might have critical effects on the amylopectin structure and the rheological properties of the starch [12]. Several floury endosperm rice mutants (*flo1*–*flo7*) have been isolated. Treating fertilized rice egg cells with the chemical mutagen *N*-methyl-*N*-nitrosourea yielded mutants *flo1* and *flo2*, which had floury endosperms [15,16]. The *flo2* mutant has been found, through map-based cloning, to be a member of the tetratricopeptide repeat-motif protein family. The gene mutation decreases the grain size and decreases the starch quality (by decreasing the amylose content) and also changes the fine structure of the amylopectin [17]. The *flo3* mutant was produced through applying gamma-irradiation and ethyl methansulfonate treatment, and had a low 16 kDa globulin content in the endosperm [18]. The white-core floury endosperm mutants *flo4* and *flo5* were produced through T-DNA insertional mutagenesis and were found to have *pyruvate orthophosphate dikinase B* and *starch synthase IIIa* (SSIIIa) gene mutations, respectively [19,20]. The *flo4* mutant endosperm had a low amylose content, suggesting that *pyruvate orthophosphate dikinase B* might play a role in regulating carbon metabolism during the grain filling process [19]. DP analysis of amylopectin in *flo5* was performed, and the amounts of DP 6–8 and DP 16–20 in the mutant endosperm were found to be decreased but the amounts of DP 9–15 and DP 22–29 had increased, indicating that *SSIIIa* strongly affects the chain-length distribution of amylopectin biosynthesized in developing rice grains [20]. The floury endosperm mutant *flo6* had a completely floury white endosperm, but the *flo7* endosperm was floury and white only at the peripheries, and not in the interior [21,22]. Map-based cloning demonstrated that *flo6* was an insertion mutation in the unknown function gene *Os03g0686900* [21] and that *flo7* was a deletion mutation in the unknown function gene *Os10g0463800* [22], suggesting that these genes may play vital roles in starch biosynthesis and granule formation in the endosperm during the grain-filling process.

Calcium ions (Ca^2+^) are secondary messengers in plant cells, and are used in response to various environmental and developmental stimuli through temporal and spatial fluctuations of the cytosolic Ca^2+^ concentration [23,24]. Calcium-dependent protein kinases (CDPKs) are a major family of calcium sensors that have been characterized in various plant species. CDPKs are Ser/Thr protein kinases that are encoded by multigene families [25,26]. CDPKs have four functional domains: an N-terminal variable domain, a catalytic kinase domain, an autoinhibitory domain, and a calcium-binding EF-hands regulatory domain [27]. Under normal growth conditions (i.e., absence of Ca^2+^ signals), the autoinhibitory domain can interact with the kinase domain thereby inhibits kinase activity [24]. Deletion of the autoinhibitory domain and the Ca^+2^ binding domains from the coding region could bypass Ca^2+^ signal stimulation and resulted in produced a constitutively active enzyme of CDPKs [26,27]. It has been shown in many studies that CDPKs play important physiological roles in response to various environmental stresses and developmental processes [23,24,28,29,30]. Only in a few studies have CDPKs been shown to play a role in starch biosynthesis during the grain-filling process in rice. The rice *SPK* (which shares 79% of its amino acid sequence with *OsCDPK1*) has been found to encode a sucrose synthase kinase. Expression of antisense *SPK* in transgenic rice produced watery grains because large amounts of sucrose had accumulated in the endosperm due to low sucrose synthase activity, resulting in inefficient sucrose degradation [31]. This indicates that SPK may be a regulator in the starch biosynthesis pathway. 

In the study presented here, *OsCDPK1* was found to play pivotal roles in rice-seed development, in the physicochemical properties of the starch produced, and in the appearance of the endosperm. The pleiotropic effects on various agronomic traits in loss- and gain-of-function of *OsCDPK1* transgenic plants are also characterized.

## 2. Results

### 2.1. Phenotypic Changes in Transgenic Rice Plants with Overexpressing or Silenced OsCDPK1

In previous studies, to understand the physiological roles of *OsCDPK1*, the gene-overexpression and gene-silencing approaches were implemented. For the truncated form of *OsCDPK1* (*OEtr*), the coding sequences in which the autoinhibitory region and the calcium-binding domains had been removed were expressed under the control of maize ubiquitin gene promoter to generate the constitutively active form of the *OEtr* transgenic plants [32]. The transgenic plants in *OEtrs* yielded smaller seeds, whereas RNA-interference gene knockdown mutants (*Ris*) yielded larger seeds [32]. In this study, the various agronomic traits in the T4 transgenic lines of *OEtrs* (*OEtr-1*, *-3* and *-4*) and *Ris* (*Ri-1*, *-2* and *-3*) were analyzed. The results (Table 1) were consistent with our previous studies [32] and showed that the *Ris* (*Ri-1*, *-2* and *-3*) lines had, on average, 7.1% and 10.8% increases in plant height and 1000-grain weight, respectively, compared to those of WT (wild type). However, both examined traits decreased in the *OEtrs* (*OEtr-1*, *-3* and *-4*) lines compared with WT, with 5.8% and 21.1% reductions in plant height and 1000-grain weight, respectively. Furthermore, compared to WT, the heading date and growth duration were shorter in *Ris*, while *OEtrs* showed no significant difference. The average of the heading date of *Ris* (83.2 day) was 6 days shorter than that of WT (89.3 day) and the growth duration of *Ris* (104.8 day) was 10 days shorter than that of WT (115.2 day). The dehulled grain weight, starch content, and amylose content were higher in *Ris* and lower in *OEtrs* than those of WT; for example, the average weights of dehulled grain of *Ris* and *OEtrs* were 111.2% and 84.1%, respectively, of that of WT. The average percentage of starch content versus dehulled grain weight in WT, *OEtrs*, and *Ris* was 73.4%, 64.2%, and 78.1%, respectively. Moreover, the average amylose content in WT, *OEtrs*, and *Ris* was 23.2%, 15.1% and 26.2%, respectively.

### 2.2. Ectopic Overexpression and Silencing of OsCDPK1 in Transgenic Rice Plants Yielded Opaque and Transparent Endosperms, Respectively

**Figure 1:**
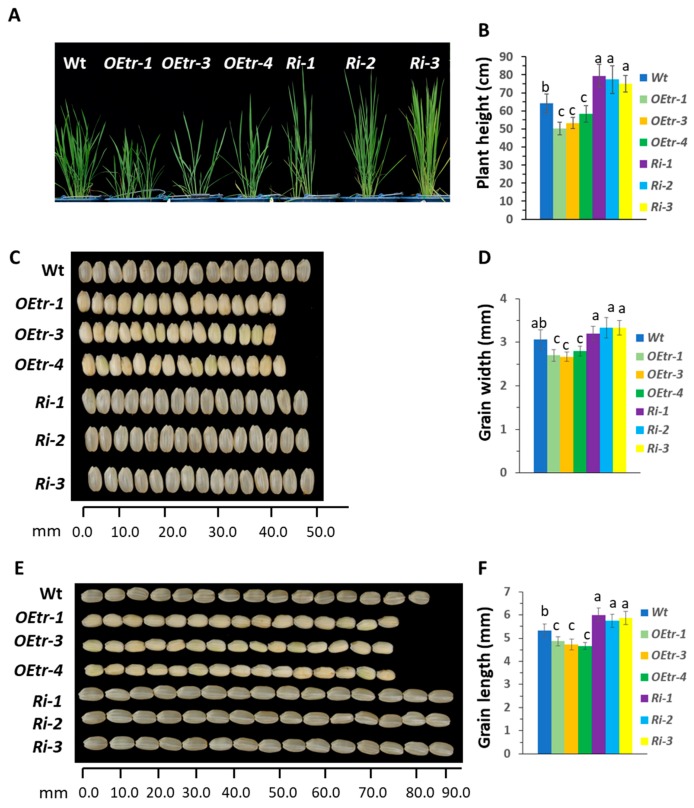
Plant heights and grain morphologies in the WT, the *OEtr-1*, *-3* and *-4* (*OEtrs*), and the *Ri-1*, *-2* and *-3* (*Ris*) plants. After 5 d of flowering, rice plants were transferred to a growth chamber and grown at an optimal temperature (25 °C for 16 h light, 20 °C for 8 h dark). (**A**) Heights of the 55-day-old WT, *OEtrs*, and *Ris* plants. (**B**) Quantification of the plant heights. Each error bar is the standard error for 15 individual plants. (**C**) Endosperm appearances and grain widths for the WT, *OEtrs*, and *Ris* plants. Fifteen grains per line were positioned in a row and measured. (**D**) Quantification of the grain widths. Each error bar is the standard error for 50 individual grains. (**E**) Endosperm appearances and grain lengths for the WT, *OEtrs*, and *Ris* plants. Fifteen grains per line were positioned in a row and measured. (**F**) Quantification of the grain lengths. Each error bar is the standard deviation (*n* = 50). Different letters above the bars indicate significant differences, identified by performing ANOVAs (*p* < 0.01).

We examined the 55-day-old plant phenotypes in the T4 transgenic lines further, and the results were consistent with Table 1. The *Ri-1*, *-2* and *-3* plants were higher (mean value is 76.6 ± 6.18 cm) and the *OEtr-1*, *-3 and -4* plants were shorter (52.4 ± 3.58 cm) than the WT plants (63.3 ± 4.63 cm) (Figure 1A,B). At the grain-filling stage, the WT and transgenic plants were transferred to the growth room at an optimal temperature of a cycle of 25 °C for 16 h light and 20 °C for 8 h dark. Fifteen seeds were randomly selected from each individual line and dehulled. We found that the dehulled *Ri-1* grains were longer and that the *OEtr-1* grains were shorter than the WT grains (Figure 1E,F). We also found that the dehulled *OEtr-1* grains were all of the floury-kernel phenotype (Figure 1C,E). 

**Figure 2:**
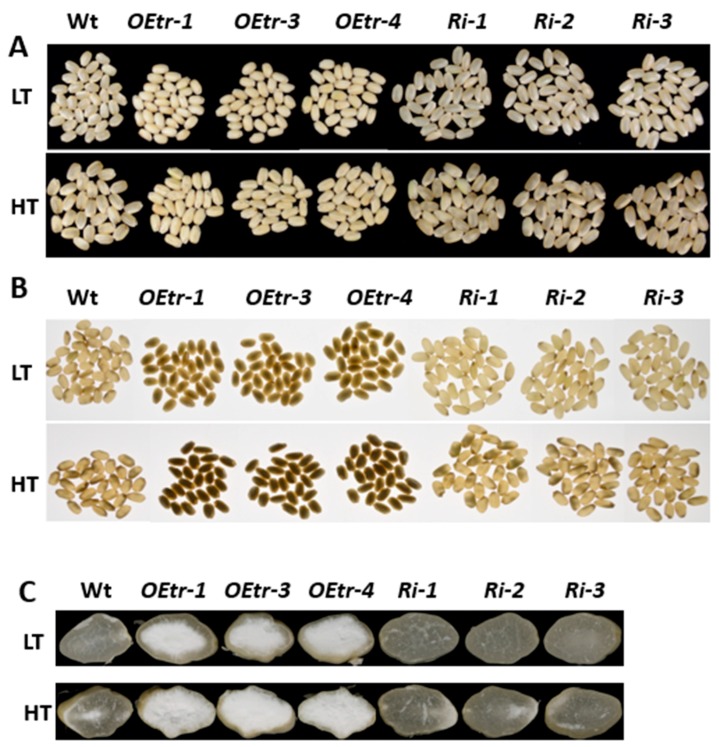
Effects of temperature on the appearances of the WT, *OEtrs*, and *Ris* endosperms. After 5 d of flowering, rice plants were transferred to a growth chamber and grown at a lower temperature (22 °C for 16 h light, 20 °C for 8 h dark) or a higher temperature (31 °C for 16 h light, 28 °C for 8 h dark). (**A**,**B**) Seeds harvested from the plants grown at the lower and higher temperatures, respectively, illuminated using (**A**) normal lighting and (**B**) backlighting. (**C**) Cross-sections of the endosperms of the seeds from plants grown at either lower or higher temperature. LT: lower temperature; HT: higher temperature.

We determined whether the temperature affected the endosperm appearance in the transgenic lines by growing rice plants at relatively low temperatures (LT; 22 °C for 16 h light, 20 °C for 8 h dark) and at relatively high temperatures (HT; 31 °C for 16 h light, 28 °C for 8 h dark) during the grain-filling process. The mature seeds were collected from the WT, the *Ri-1*, *Ri-2* and *Ri-3* (*Ris*), and the *OEtr-1*, *OEtr-3* and *OEtr-4* (*OEtrs*) plants. As shown in Figure 2, under LT and HT conditions, all the dehulled *OEtrs* grains had smaller and floury endosperms (100% in the *OEtr-1*, *OEtr-3* and *OEtr-4* lines). In contrast, under LT condition, most of the grains in the WT and *Ris* lines displayed transparency phenotype; the ratios of chalky grains were 16.2% in the WT grains, and 6.0%, 6.7%, and 7.1% in the *Ri-1*, *Ri-2* and *Ri-3* grains, respectively (Figure 2A and Table 2). However, the higher temperature caused a significant increase in the WT and *Ris* lines’ endosperms of the chalky phenotype; the ratios of chalky grains were 63.6% in the WT grains, and 50.1%, 51.2% and 45.1% in the *Ri-1*, *Ri-2* and *Ri-3* grains, respectively (Figure 2A and Table 2). Illuminating the kernels with a backlight showed that the *OEtrs* grains all had opaque endosperms regardless of the temperature at which the plants were grown at LT or HT (Figure 2B). However, most of the WT and *Ris* grains displayed transparent endosperms under the LT condition (the *Ris* endosperms being more transparent than those of WT), the proportion of opaque endosperms increased to more than 50% in both the WT and *Ris* lines under HT growth condition (Figure 2B and Figure S1). Cross-sections of the endosperms (Figure 2C) indicated that the kernels in the WT and *Ris* displayed transparent phenotype under LT treatment and showed partial opaque phenotype under HT condition. However, in the *OEtrs*, all endosperms displayed completely floury appearance under both LT and HT conditions, regardless of the different temperature treatments used. These results suggest that *OsCDPK1* affects rice endosperm appearance in a temperature-independent manner.

### 2.3. Effect of OsCDPK1 on Starch Granule Morphology in Rice Endosperms

**Figure 3:**
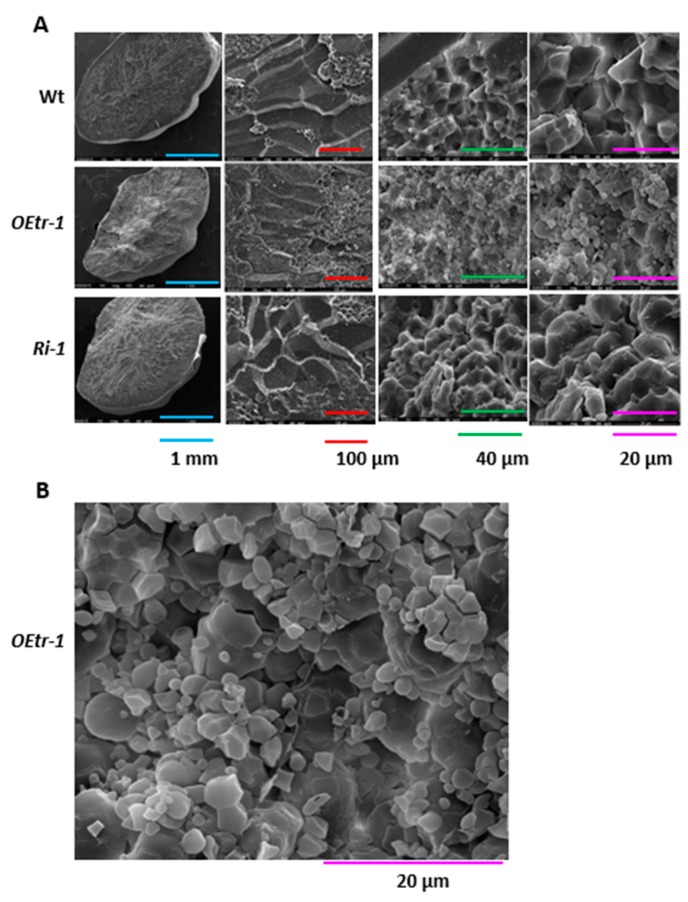
Scanning electron microscopy images of the structures of the starch granules in the rice endosperms. (**A**) The central areas of the cross-sections of mature endosperms from the WT plants (top panel), *OEtr-1* plants (middle panel), and *Ri-1* plants (bottom panel) were acquired using a scanning electron microscope. (**B**) A zoomed-in image of the 20 μm photo of *OEtr-1*. Scale bar colors: blue as 1 mm, red as 100 μm, green as 40 μm, purple as 20 μm.

Due to the similar transgenic plant phenotypes and grain morphology (Figure 1 and Figure 2), the *OsCDPK1*-overexpressing line, *OEtr-1*, and the *OsCDPK1*-silencing line, *Ri-1*, were therefore selected for further study. We examined the starch granule morphology in the WT, *Ri-1*, and *OEtr-1* transgenic seeds by analyzing the endosperm cross-sections acquired from scanning electron microscopy. As shown in Figure 3, the three-dimensional structures of the starch granules in the endosperms were irregularly polygonal and polyhedral in all three grains types. The starch granules were large and tightly packed in the WT and *Ri-1* endosperms but small and loosely packed in the *OEtr-1* endosperm (Figure 3B), suggesting that *OsCDPK1* affects the starch granule size and packing density in developing rice seed.

### 2.4. Effects of OsCDPK1 on Starch Properties and Gelatinization in the Endosperm

**Figure 4:**
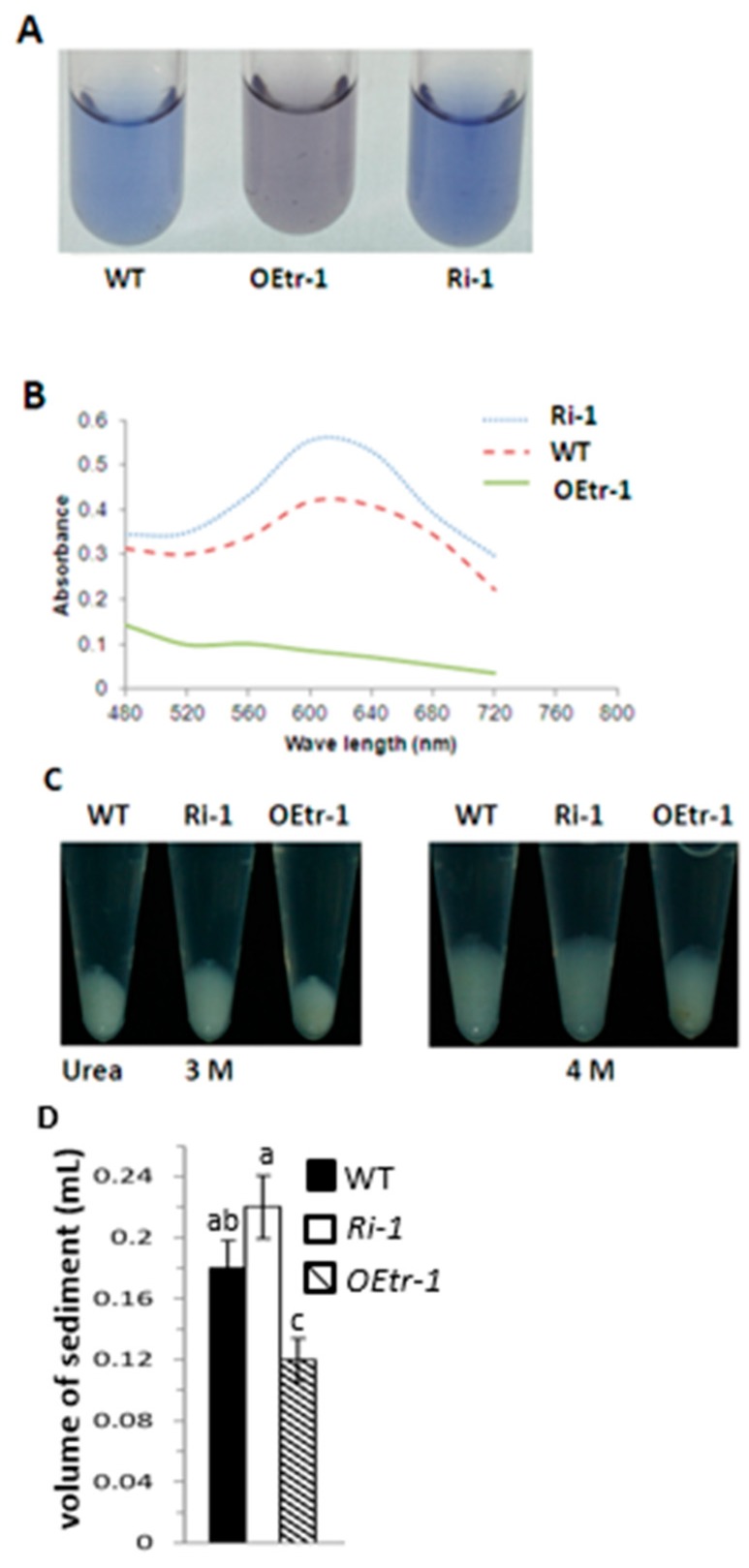
Iodine-staining and gelatinization properties of the starch in the rice endosperms. A 20-mg aliquot of endosperm powder was treated with 1 N NaOH as described in Section 4. (**A**) Supernatants of the iodine-stained WT, *OEtr-1*, and *Ri-1* samples. (**B**) Starch–iodine absorbance spectra of the supernatants. (**C**) Effects of using 3.0 and 4.0 M urea solutions on the gelatinization characteristics of the WT, *OEtr-1*, and *Ri-1* endosperm starch. A 20-mg aliquot of rice powder was mixed in an Eppendorf tube with 1 mL of urea solution and the mixture was shaken for 24 h at 25 °C. The mixture was centrifuged, and the volume of the gelatinized starch sediment was measured. (**D**) Quantification of the gelatinization volume. Different letters above the bars indicate significant differences, identified by performing ANOVAs (*p* < 0.05). Each value is the mean ± SD of three independent measurements.

We examined the apparent amylose content in the endosperm by reacting 20 mg of each rice endosperm powder sample with 1 N NaOH to gelatinize the starch. The amylose content was measured using a colorimetric method using an I_2_/KI solution [12,33]. The *OEtr-1* samples had less affinity than the other samples for iodine and were light purple, whereas the *Ri-1* and WT samples were dark blue and light blue, respectively (Figure 4A and Figure S2). As described in Table 1, the average amylose content in WT, *OEtrs*, and *Ris* was 23.2%, 15.1% and 26.2%, respectively. Here, the apparent amylose content in WT, *OEtr-1* and *Ri-1* were examined in detail to dissect the roles of *OsCDPK1* in starch biosynthesis. The absorption spectra of the I_2_/KI stained solutions and found strong absorbance between 480 and 720 nm for both the *Ri-1* and WT samples, with maxima at 620 nm, but stronger absorbance was found for *Ri-1* than for WT at the same wavelength (Figure 4B). *OEtr-1* absorbance was weaker and decreased as the wavelength increased. We compared the results to the absorbances of potato amylose standards to allow the apparent amylose contents to be determined. The apparent amylose contents of the WT, *OEtr-1*, and *Ri-1* samples were 23.35%, 14.74%, and 26.15%, respectively (Table S2). These results demonstrate that the *Ri-1* seed endosperms had higher amylose content than the WT seed endosperms and that the *OEtr-1* seed endosperms had lower amylose content than the WT seed endosperms. Because the WT, *OEtr-1*, and *Ri-1* amylose contents were different, we investigated starch gelatinization at different urea concentrations (0–9 M). A 20-mg aliquot of a rice endosperm powder was mixed with 1 mL of urea solution, and the mixture was allowed to react for 24 h. The mixture was then centrifuged and the degree of gelatinization determined by measuring the sediment volume. Starch gelatinization started at urea concentrations of 3.0–4.0 M (Figure 4C and Figure S3). In 4.0 M urea, the *Ri-1* sediment volume was 5.3% higher than the WT sediment volume, whereas the *OEtr-1* sediment volume was 36.4% lower than the WT sediment volume (Figure 4D). These results indicate that *OsCDPK1* affects the physicochemical properties of the starch in rice endosperms.

### 2.5. OsCDPK1 Expression Profiles in Developing Rice Seeds

**Figure 5:**
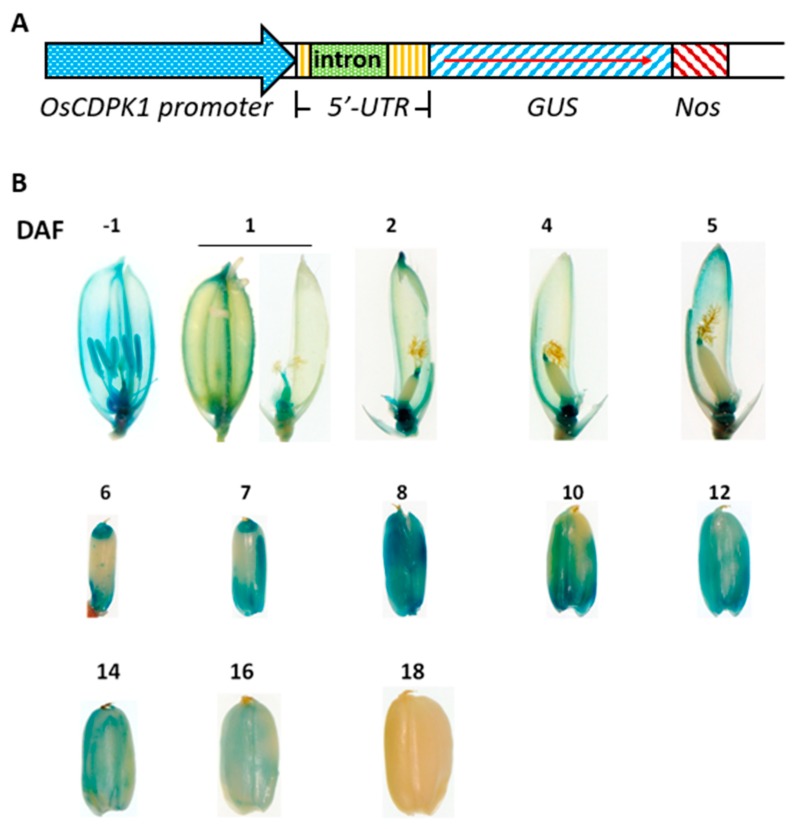
Histochemical GUS activity staining in flowers and developing rice seeds. (**A**) Map of the *OsCDPK1::GUS* expression construct. (**B**) Rice spikelets were collected before flowering and 1–18 days after flowering (DAF). The lemma and palea were partially or completed removed from each spikelet or developing seed before staining. The stained spikelets or immature grains were preserved in 70% ethanol and photographed. −1 DAF means before flowering.

Our results have demonstrated that *OsCDPK1* affects rice seed development. It is necessary to track changes in *OsCDPK1* gene expression during rice seed development. The *OsCDPK1::GUS* transgenic line was generated using a *GUS* (*β-glucuronidase*) reporter gene controlled by the *OsCDPK1* promoter (−1706 to +301 bp, i.e., a total of 2007 bp upstream of the translational start site) containing the first intron (607 bp, in the 5′-untranslated region) (Figure 5A). As shown in Figure 5B, strong GUS staining was observed in the ovaries and anthers before flowering, but weaker staining was observed in the styles and lemma. At 1 DAF (days after flowering), strong GUS activity was found only in the ovaries and styles, weaker activity was found in the lemma, and no GUS activity was found in the anthers and stigma. Between 2 and 5 DAF, concentrated GUS staining was found in the rachilla and both ends of the developing seeds and weak staining was found in the lemma. The blue color gradually expanded from both ends toward the central parts of the developing seeds between 6 and 7 DAF, and at 8 DAF the entire seeds were thoroughly stained blue. Staining gradually decreased afterwards, but remained strong between 10 and 14 DAF, then decreased quickly after 14 DAF and had completely gone by 18 DAF. These results suggest that *OsCDPK1* was expressed in a particular temporal and spatial way, predominantly in the middle stage of rice seed development.

### 2.6. Effects of OsCDPK1 on the Levels of Starch-Biosynthesis-Related Genes in Developing Rice Seeds

**Figure 6:**
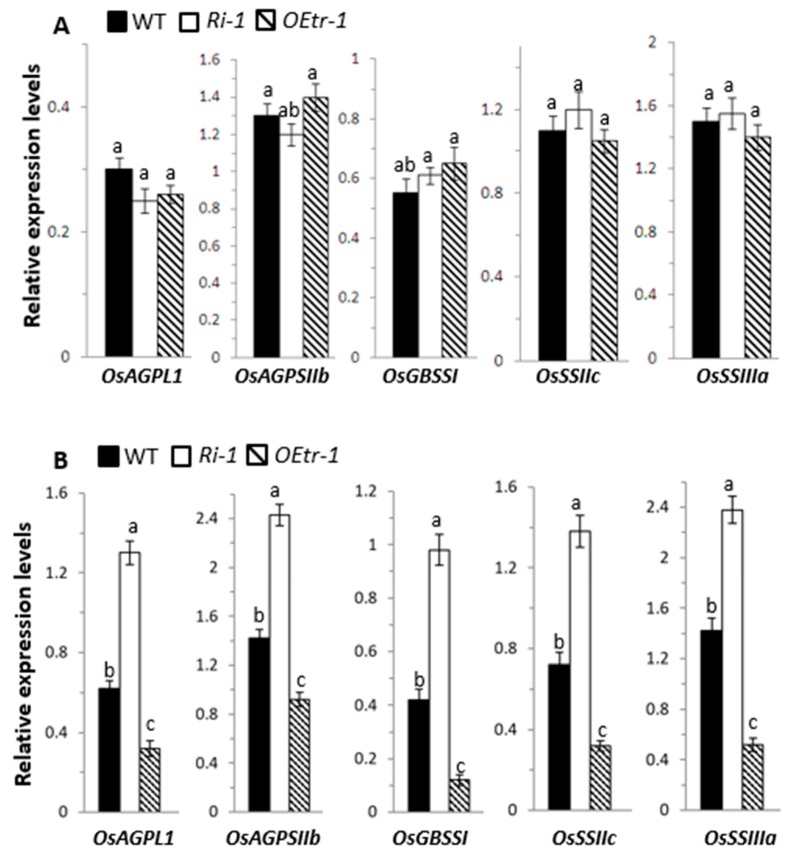
Expression of starch-biosynthesis-related genes during seed development in the WT, *Ri-1*, and *OEtr-1* plants. Total RNA was isolated from developing endosperms (**A**) 5 and (**B**) 12 DAF from the WT, *Ri-1*, and *OEtr-1* plants, and subjected to RT-PCR analysis. The relative expression levels of each gene were normalized to the expression level of the internal control *OsActin*. Different letters above the bars indicate significant differences, identified by performing ANOVAs (*p* < 0.01). Each value is the mean ± SD of three independent measurements. *OsAGPLI*: ADP-glucose pyrophosphorylase large subunit I; *OsAGPSIIb*: ADP-glucose pyrophosphorylase small subunit IIb; *OsGBSSI:* granule-bound starch synthase I; *OsSSIIc:* starch synthase IIc; *OsSSIIIa:* starch synthase IIIa. Primer sets and gene accession numbers are listed in Table S1.

We further investigated the roles of *OsCDPK1* in rice-seed development by analyzing the expression patterns of 12 genes involved in starch biosynthesis. These genes were granule-bound starch synthase (*OsGBSSI*), starch synthase (*OsSSI*, *OsSSIIa*, *OsSSIIb*, *OsSSIIc*, *OsSSIIIa*, and *OsSSIIIb*), branching enzyme (*OsBEI*), ADP-glucose pyrophosphorylase large subunit (*OsAGPLI*, *OsAGPLII*, and *OsAGPLIII*), and a small subunit of ADP-glucose pyrophosphorylase (O*sAGPSIIb*). The developing WT, *Ri-1*, and *OEtr-1* rice seeds at 5 and 12 DAF were analyzed. Total RNA was isolated from the dehulled embryo-less half seeds and subjected to quantitative RT-PCR. The relative expression levels of *OsAGPLI*, *OsAGPSIIb*, *OsGBSSI*, *OsSSIIc*, and *OsSSIIIa* were significantly up-regulated in *Ri-1* and down-regulated in *OEtr-1* at 12 DAF, but there was no significant difference at 5 DAF compared with the genes in WT plants (Figure 6). Expression of the seven other genes in the transgenic lines was not significantly different at 5 or 12 DAF from expression in the WT plants. These results suggest that *OsCDPK1* might be involved in regulating starch-biosynthesis-related genes in the mid-development stage of the rice seed.

## 3. Discussion

The amylose content of endosperms is an important parameter determining the eating quality of rice, which is negatively related with stickiness but positively related to rice grain hardness [34,35]. The key enzymes (genes) involved in the starch (amylopectin and amylose) biosynthesis during rice grain filling are well known. However, the control mechanisms by which the amylose and amylopectin biosynthesis is orchestrated are still not fully understood. Our results indicate for the first time that the protein kinase *OsCDPK1* is functionally negatively correlated with the amylose content, endosperm transparency, and seed size in developing rice seed.

The *OEtr-1* and *Ri-1* seeds had some distinct features compared with the WT seeds. For example, the *OEtr-1* grains were smaller, had lower amylose contents, and had more floury endosperms than the WT grains, and the *Ri-*1 grains were larger, had higher amylose contents, and had more transparent endosperms than the WT grains (Figure 1, Figure 2, Figure 3 and Figure 4). This indicates that the *OsCDPK1* function is closely associated with the rice endosperm starch properties. The *OsCDPK1::GUS* expression profile in the developing rice grains gradually increased immediately after flowering and reached a maximum between 7 and 14 DAF (Figure 5). The *OsCDPK1::GUS* expression timing in the developing rice seeds was similar to that found in a study by Ohdan et al. [36], in which 27 different genes involved in starch biosynthesis were examined during rice-seed development. In that study, all the genes had been differentially expressed before 15 DAF. In this study, the GUS staining was found throughout the endosperm at 12 and 14 DAF of developing seeds, and was more intensified in the interior region at 12 DAF (Figure S4), suggesting that *OsCDPK1* first affects the expression of starch biosynthesis-related genes and later affects the starch composition and endosperm appearance. The starchy endosperm in the *OEtr-1* grains had a low amylose content and an opaque floury appearance, and the starch granules were small and loosely packed (Figure 1, Figure 2 and Figure 3), indicating that the *OsCDPK1* roles were closely associated with the structures and qualities of the starch granules during the grain filling process. Moreover, *OsCDPK1::GUS* staining was found throughout the developing endosperm at 7–16 DAF (Figure 5B and Figure S4). These results suggest that the expression of some starch-biosynthesis-related genes in the endosperm cells may be affected by *OsCDPK1*, followed by changing the amylose content and resulting in the opaque endosperm in the *OEtr-1* grains.

Several mutations of rice genes involved in starch biosynthesis have been found to alter the structures and properties of the starch produced. For example, the *SSIIIa* mutation (*flo5*) was found to increase the amylose content, alter the amylopectin structure, and cause the endosperm to have a white core [20]. The *waxy* mutant (a mutation in *GBSSI*) produced an amylose-free, floury endosperm [11,12]. The *amylose-extender* mutation in *BEIIb* altered the fine structure of amylopectin and gave a floury endosperm [12]. A mutation in *BEI* (starch-branching enzyme I) caused the amylopectin fine structure to change but did not appear to affect the endosperm appearance [10]. Our data show that the expression of some starch-biosynthesis-related genes was affected by *OsCDPK1*, similar to the results of previous studies. During the middle phase (12 DAF) of endosperm development, examples of genes affected were *OsAGPLI*, *OsAGPSIIb*, *OsGBSSI*, *OsSSIIc*, and *OsSSIIIa*, which were significantly up-regulated in the *Ri-1* and down-regulated in *OEtr-1* (Figure 6). Changes in the expression of these genes caused the *OEtr-1* endosperm to have a lower amylose content and a floury appearance, whereas the *Ri-1* endosperm had a high amylose content and was more transparent (Figure 1, Figure 2 and Figure 4 and Table 1 and Table 2). Calcium ions (Ca^2+^) are secondary messengers in plant cells, with plant CDPK being a sensor to relay calcium signals via binding with calcium via the calcium-binding domains. We therefore suggest that *OsCDPK1* acts as an upstream regulator that is closely associated with starch biosynthesis. Some regulators have been found to regulate the expression of genes encoding key starch-biosynthesis enzymes, and null mutations in these regulators usually alter the amylopectin fine structure, the starch composition, the starch granule morphology and size, and the endosperm appearance. For instance, some starch-biosynthesis-related genes were found to be affected in five independent mutants, *flo2* (a tetratricopeptide repeat motif protein) [17], *flo4* (*pyruvate orthophosphate dikinase B*) [19], *flo7* (an unknown protein) [22], *osbzip58* (a bZIP transcription factor) [37], and *osbt1* (an ADP-glucose transporter) [38]. All these effects decreased the amylose content and changed the amylopectin composition. The endosperm appearance was affected differently in different parts; e.g., *flo2* had endosperm with a floury kernel, *flo4* and *osbzip58* and *osbt1* gave white-core phenotypes, and chalkiness was only found in the peripheral endosperm of *flo7*. Similarly, the low amylose content of the *OEtr-1* endosperm was expressed in a floury morphology (Figure 1 and Figure 4). The results of previous studies and this study together indicate that a low-amylose content of rice endosperm gives a chalky or floury phenotype, suggesting that the amount of amylose present is an important factor affecting the quality and appearance of the starchy endosperm. This raises the question of whether rice can be engineered to have an endosperm with a high amylose content and, therefore, a transparent appearance. Here, we have provided direct evidence that silencing *OsCDPK1* (*Ri-1*) increases the amylose content of the endosperm, making the starchy grain more transparent (Figure 1 and Figure 4 and Table 1 and Table 2). These results will be useful in developing rice-breeding strategies aimed at maintaining (or even improving) grain quality in rice to cope with global warming. The transgenic lines *OEtrs* and *Ris* could also be ideal materials for investigating the mechanisms that control rice seed size and starch biosynthesis.

Temperature also strongly affects amylose synthesis during rice grain development. In previous studies, a lower temperature increased the expression of the *waxy* gene and protein and increased the amylose content in developing rice endosperms [39]; a higher temperature had the opposite effects [39,40]. Moreover, during the rice grain-filling process, a temperature higher than the optimum usually causes impaired starch accumulation, resulting in loosely packed starch granules with small air spaces between them, giving high proportions of opaque chalky or floury grains. These effects decrease the market value because the rice will have poor milling qualities (being easily broken), poor cooking and eating qualities, and a poor appearance [41,42]. It has recently been found that a high temperature (33 °C for 12 h light, 28 °C for 12 h dark) also induced the expression of three α-amylase genes—*Amy1A*, *Amy3C*, and *Amy3D*—in developing endosperms, causing the grains to be chalky, probably because of starch degradation and the accumulation of soluble sugars in the endosperm [43]. In contrast, we found that the *Ri-1* endosperm was more transparent at both a low temperature (22 °C for 16 h light, 20 °C for 8 h dark) and a high temperature (31 °C for 16 h light, 28 °C for 8 h dark) than that of the WT plant during the grain-filling process (Figure 2 and Table 2). Our results will be useful in developing rice-breeding strategies aimed at maintaining (or even improving) grain quality in rice selected to cope with global warming. Our results also improve our understanding of the molecular mechanisms involved in amylose biosynthesis.

It has been found in several studies that chalky or floury endosperms might be caused by loosely packed small, round starch granules formed in developing rice seeds [9,19,20,38]. The scanning electron microscopy images indicate that the WT and *Ri-1* endosperms contained closely packed polyhedral starch granules but that the white core of the *OEtr-1* endosperm contained loosely packed small starch granules. The starch granules in the *OEtr-1* endosperm were polyhedral (like in the WT and *Ri-1* endosperm) rather than small and round as in most chalky or floury mutants (Figure 3B). It is, therefore, likely that *OsCDPK1* plays a role in starch biosynthesis and negatively affects the sizes but not the shapes of the starch granules. 

**Figure 7:**
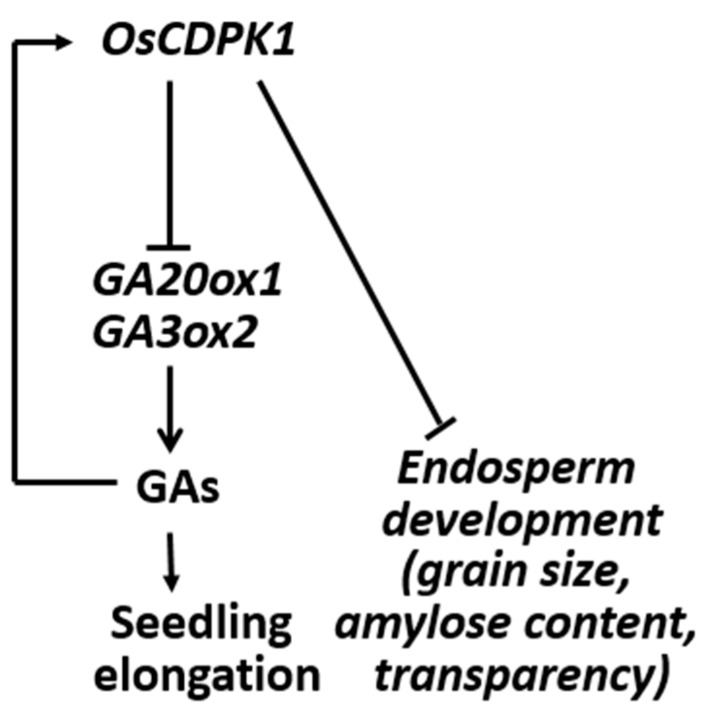
Proposed roles of *OsCDPK1* in the interconnecting GA biosynthesis and signaling pathways and endosperm developmental processes. The model is described in detail in the text. The arrows indicate activation and the blunt ends indicate inhibition.

We previously found that *OsCDPK1* inhibits the feedback of GA biosynthesis through down-regulating *GA3ox2* and *GA20ox1* [32]. In this study, we put forward a model in which *OsCDPK1* plays key roles in negatively controlling the grain size, amylose content, and endosperm appearance, and also affects the physicochemical properties of the starch (Figure 7). Milled *OsCDPK1-*gene-silenced *Ri-1* grains were larger than WT grains, and there were numerous densely packed polyhedral starch granules accompanied by a high amylose content and a transparent endosperm. In contrast, the *OEtr-1* grains were smaller and contained loosely packed small, but still polyhedral, starch granules. The *OEtr-1* grains had lower amylose content and opaque white-cored endosperms. Notably, the phenotypes of the grain size and the floury endosperm in *OEtr-1*, *-2*, and *-3* were consistent and were unaffected by temperature during the grain-filling process (Figure 1 and Figure 2). *OsCDPK1* therefore plays pleiotropic roles in rice seed development. Our results indicate that *Ri-1* and *OEtr-1* could be ideal materials for investigating the mechanisms controlling rice seed size and starch biosynthesis, and could also be valuable reference samples for rice breeding aimed at simultaneously improving grain yield and quality.

## 4. Materials and Methods

### 4.1. Plant Materials

In our previous studies [32], 8 independent T1 transgenic lines that carried a single transgene in rice were subjected to ectopic overexpression of a constitutively active truncated form of *OsCDPK1* (*OEtr*) in rice seedlings, which all yielded a semi-dwarf phenotype that produced small seeds. By contrast, 5 independent T1 transgenic plants exhibiting a single copy of transgene were subject to *OsCDPK1* gene silencing (*Ri*) by RNA interference, which all gave a slender-like phenotype during seedling development and subsequently produced large seeds. According to the consistency of the phenotypic traits in *OEtr* lines of which all displayed semi-dwarf phenotype and produced small seeds. In *Ri* lines, which all gave a slender-like phenotype and produced large seeds [32]. Therefore, in this study, the homozygous transgenes of the T4 transgenic plants from *OEtr-1*, *OEtr-3*, and *OEtr-4*, and *Ri-1*, *Ri-2*, and *Ri-3* were selected to explore the roles of *OsCDPK1* in rice grain development.

### 4.2. Callus Induction

Rice *Oryza sativa* L. cv. Tainung 67 was used in the study. Immature seeds were de-hulled, sterilized with 2.4% NaOCl for 30 min, then washed thoroughly with sterile water. The seeds were then incubated on N6 agar medium [44] containing 10 µM 2,4-D to induce calli formation [45]. After about 30 days, the calli derived from the scutella were transferred to the fresh N6 agar medium containing 2,4-D for another 15 days and were subjected to *Agrobacterium*-mediated gene transformation [45]. 

### 4.3. Primers

The nucleotide sequences of all the primers used in the real-time polymerase chain reaction (qRT-PCR) amplification are shown in Table S1.

### 4.4. Construction of OsCDPK1::GUS Expression Vectors

The *OsCDPK1::GUS* expression vector was constructed by amplifying a 2007 bp DNA fragment containing the *OsCDPK1* promoter and its 5′-untranslated region (which contained a 607 bp intron) (Figure 1A) by PCR using the forward primer *OsCDPK1-5P* (5′-ATACTGCAGTGGTCTTATT AGGTAAGGCC-3′) and the reverse primer *OsCDPK1-3B* (5′-ATAGGATCC TCCAAGAACTCCTTATGCAA-3′). The DNA fragment was cleaved using *Pst*I and *Bam*HI, and then cloned into vector *pBX-2* as described previously [45]. The *OsCDPK1::GUS* construct was linearized by digesting it with *Pst*I, then inserted into the *Pst*I site of the *pSMY1H* binary vector [45], then it was subjected to *Agrobacterium*-mediated gene transformation.

### 4.5. Plant Transformation

Recombinant binary plasmids were introduced into *Agrobacterium tumefaciens* strain EHA101 by electroporation, and rice calli were transformed as described previously [45].

### 4.6. Histochemical Staining of GUS Activity in Developing Rice Grains 

Rice spikelets collected before flowering and 1–18 DAF were subjected to GUS activity staining to assess *OsCDPK1* gene expression profiles in developing rice grains. The lemma and palea were partially or completely removed from each spikelet (developing seed) before the staining process, and the spikelets were then incubated in a 1-mM 5-bromo-4-chloro-3-indolyl β-D-glucuronide solution (in 100 mM sodium phosphate containing 10 mM EDTA, 0.5 mM potassium ferrocyanide, 0.5 mM potassium ferricyanide, and 0.1% Triton X-100, at pH 7.0) at 37 °C in the dark for 4 h. The stained spikelets or immature grains were then preserved in 70% ethanol and rinsed with water before being photographed.

### 4.7. Quantitative RT-PCR

Developing seeds were collected from wild-type (WT), *Ri-1*, and *OEtr-1* at 5 and 12 DAF, respectively. Total RNA was isolated from developing endosperms by using TRIzol reagent (Invitrogen, Carlsbad, CA, USA), and DNA contamination was then removed using a TURBO DNA-free kit (Ambion, Foster City, CA, USA). A 5-µg aliquot of the total RNA was used to synthesize first strand cDNA using M-MuLV reverse transcriptase (New England Biolabs, Ipswich, MA, USA) and oligo (dT) primer. Quantitative RT-PCR was performed using an Eco Real-Time PCR System (Illumina, San Diego, CA, USA) following the manufacturer’s instructions. Gene-specific primer sets (Table S1) localized at the 3′-untranslated regions for each gene examined were selected to allow assessment of the extent to which the starch-biosynthesis-related genes in WT, *OEtr-1*, and *Ri-1* were expressed. The relative expression levels were normalized to expression in the internal control, *OsActin 1*. All reactions were performed in triplicate.

### 4.8. Analysis of the Gelatinization Properties of the Starch

A 20-mg aliquot of rice endosperm powder derived from de-embryonic seeds was mixed with 1 mL of urea solution at a concentration of between 0 and 9 M, and the mixture was shaken vigorously for 24 h at room temperature [12]. All the tested mixtures were then centrifuged at 25,000× *g* for 60 min (Eppendorf, model 5427R, North Ryde, Australia) at a same time, and the volume of the gelatinized starch sediment was measured by the volume scale on the Eppendorf tube.

### 4.9. Apparent Amylose Content Analysis

A 20-mg aliquot of rice endosperm powder was gelatinized by adding 2 mL of 1 N NaOH and incubating the mixture at 25 °C for 24 h. Then, 4 mL of 1 N CH_3_COOH was added, the mixture was mixed well, and 4 mL H_2_O was added. A 0.8-mL aliquot of the solution was mixed with 0.2 mL I_2_/KI (0.2%/2%), then 4 mL H_2_O was added. The apparent amylose content was measured using the colorimetric method described by [33]. Absorbance at 620 nm was measured, and the apparent amylose content was determined by comparing the absorbance to a calibration curve prepared using potato amylose standards.

### 4.10. Scanning Electron Microscopy 

Dehusked rice grains were cut transversely and analyzed using a scanning electron microscope (Quanta 200; FEI, Hillsboro, OR, USA) following the manufacturer’s instructions.

## 5. Conclusions

Our results demonstrated that *OsCDPK1* plays key roles in negatively controlling the grain size, amylose content, and endosperm appearance, and also affects the physicochemical properties of the starch. Milled *OsCDPK1*-gene-silenced *Ri-1* grains were larger than WT grains, and there were numerous densely packed polyhedral starch granules accompanied by a high amylose content and a transparent endosperm. In contrast, the *OEtr-1* grains were smaller and contained loosely packed small, but still polyhedral, starch granules. The *OEtr-1* grains had lower amylose content and opaque white-cored endosperms. Moreover, the grain phenotypes in *OEtrs* were unaffected by temperature during the grain-filling process. *OsCDPK1* therefore plays pleiotropic roles in rice reproductive developmental processes, in a negative sense. Our results indicate that *Ri-1* and *OEtr-1* could be ideal materials for investigating the mechanisms that control rice seed size and starch biosynthesis, and for rice breeding to improve grain yield and quality.

## Figures and Tables

**Table 1:** Comparison of agronomic traits between WT and transgenic lines.

Genotypes	Plant Height (cm)	Heading Day (day)	Growth Duration (day)	1000-Grain (g)	Dehulled Grain (mg/grain)	Starch Content (mg/grain)	Amylose Content (%)
WT	108.2 ± 2.5	89.3 ± 2.4	115.2 ± 3.3	23.2 ± 0.6	21.4 ± 0.3	15.7 ± 0.4 (73.4%) ^a^	23.2 ± 0.3
*OEtr-1*	99.5 ± 3.5 *	92.1 ± 2.3	117.6 ± 2.7	18.2 ± 0.3 **	16.8 ± 0.2 **	10.5 ± 0.1 ** (62.5%) ^a^	14.3 ± 0.3 *
*OEtr-3*	102.6 ± 2.6 *	92.4 ± 3.4 *	119.1 ± 3.8	18.6 ± 0.1 **	17.3 ± 0.4 **	11.8 ± 0.3 ** (68.2%) ^a^	15.3 ± 0.2 *
*OEtr-4*	103. 6 ± 3.1 *	91.1 ± 2.7 *	119.4 ± 3.3	18.1 ± 0.3 **	16.6 ± 0.2 **	10.3 ± 0.1 ** (62.0%)^a^	15.6 ± 0.4 *
*Ri-1*	115.4 ± 4.6 *	83.6 ± 2.5 **	106.6 ± 3.1 *	25.6 ± 0.3 *	23.9 ± 0.3 *	18.7 ± 0.2 * (78.2%)^a^	25.8 ± 0.1 *
*Ri-2*	118.7 ± 3.8 *	82.7 ± 2.8 **	103.6 ± 2.1 *	25.3 ± 0.2 *	23.5 ± 0.2 *	18.5 ± 0.2 * (78.7%) ^a^	26.6 ± 0.5 *
*Ri-3*	113.6 ± 4.3 *	83.2 ± 3.4 **	104.3 ± 3.6 *	26.2 ± 0.5 *	24.4 ± 0.2 *	18.9 ± 0.6 * (77.5%) ^a^	26.2 ± 0.2 *

Mean values calculated from three independent transgenic lines. All data are presented as mean ± SE. Statistical significance is determined by *t*-test. Values in the same column indicate significant differences between WT and mutant lines at * *p* < 0.05 and ** *p* < 0.01. ^a^ Percentage of dehulled grain weight.

**Table 2:** The ratios of chalky grains in wild type (TNG67), *OEtr-1*, and *Ri-1* lines growth under lower temperature (LT; 22 °C for 16 h light, 20 °C for 8 h dark) or higher temperature (HT; 31 °C for 16 h light, 28 °C for 8 h dark) during the rice grain-filling process.

Plant Species	Wild Type	*OEtr-1*	*OEtr-3*	*OEtr-4*	*Ri-1*	*Ri-2*	*Ri-3*
The ratios of chalky grains in LT (%)	16.2 ± 2.19	100	100	100	6.0 ± 0.61	6.7 ± 0.56	7.1 ± 0. 61
The ratios of chalky grains in HT (%)	63.6 ± 5.19	100	100	100	50.1 ± 3.61	51.2 ± 4.56	45.1 ± 3.21

Mean values calculated from 100 independent seeds. All data are presented as mean ± SE. Statistical significance is determined by *t*-test.

## Supplementary Materials

The following are available online at http://www.mdpi.com/1422-0067/19/10/3247/s1.
