# Supplementary material for: Effects of OsCDPK1 on the Structure and Physicochemical Properties of Starch in Developing Rice Seeds
Source: Int J Mol Sci. 2018 Oct 19;19(10):3247. doi: 10.3390/ijms19103247 (PMC6214144; doi:10.3390/ijms19103247)
Supplement: Supplementary file 1 [file ijms-19-03247-s001.zip › ijms-358717-SI.pdf]

Int. J. Mol. Sci. 2018, 19, 3247; doi:10.3390/ijms19103247 www.mdpi.com/journal/ijms

Supplementary information

# Effects of OsCDPK1 on the Structure and Physicochemical Properties of Starch in Developing Rice Seeds

Jian-Zhi Jiang[1], Chun-Hsiang Kuo[1], Bo-Hong Chen[1], Mao-Kei Chen[1], Choun-Sea Lin[2], Shin-Lon Ho[1]*

[1]Department of Agronomy, National Chiayi University, Chiayi 60004, Taiwan, Republic of China
[2]Agricultural Biotechnology Research Center, Academia Sinica, Taipei, Taiwan, Republic of China

*Corresponding author
Shin-Lon Ho
Tel.: +886-5-2717388
Fax: +886-5-2717386,
E-mail: slho@mail.ncyu.edu.tw

**Table S1** Primers used in real time RT-PCR

| Name | Sequence |
|---|---|
| *OsAGPLI*-RT5 | 5'-CGCTGATGCC ACAGATCTCT-3' |
| *OsAGPLI*-RT3 | 5'-GCCATGTGGAATCAACGAGC-3' |
| *OsAGPLII*-RT5 | 5'- GCATAGATAGGCCTTGGAAT -3' |
| *OsAGPLII*-RT3 | 5'-TACGCTATCCGTCTTGAATC -3' |
| *OsAGPLIII*-RT5 | 5'-CAGCCATGACCATTTGCGGC-3' |
| *OsAGPLIII*-RT3 | 5'-TAGATTCACGAGACAATGAT-3' |
| *OsAGPSIIb*-RT5 | 5'- GCAACCAATCACCACTGTAC-3' |
| *OsAGPSIIb*-RT3 | 5'- GGCAGCATGGAATAAACCAC-3' |
| *OsGBSSI*-RT5 | 5'-AGATCTACATATGGAGTGAT -3' |
| *OsGBSSI*-RT3 | 5'-GCCTATATAATTGGCTATAG -3' |
| *OsSBEI*-RT5 | 5'-GATTTCTTGATCAGGAGCAA -3' |
| *OsSBEI*-RT3 | 5'-ATAGGTACATAGCAACTGCT-3' |
| *OsSSI*-RT5 | 5'-ATCAACCATATGTCATGTAA-3' |
| *OsSSI*-RT3 | 5'-GTACAAAGTTTTCATTCCGC-3' |
| *OsSSIIa*-RT5 | 5'-TATAGCTATAGCCTCCCTGA-3' |
| *OsSSIIa*-RT3 | 5'-TCAACGCACAGTACGGTCAG -3' |
| *OsSSIIb*-RT5 | 5'-CCTCTGGATCCCGCCGTGGA-3' |
| *OsSSIIb*-RT3 | 5'- GCAGCGTGCCAGTCATCGTG-3' |
| *OsSSIIc*-RT5 | 5'- CTCGACGGTTGTTCAGTCAT-3' |
| *OsSSIIc*-RT3 | 5'-CACAGGAAGATGTAAGCCAT-3' |
| *OsSSIIIa*-RT5 | 5'- AACGGTGGCAAGAGAAAGCA -3' |
| *OsSSIIIa*-RT3 | 5'- GTTAATTTGTATCTGCAGCT -3' |
| *OsSSIIIb*-RT5 | 5'- ACACATGGATTTTGGGAAGT -3' |
| *OsSSIIIb*-RT3 | 5'-TTCTTTCTGTCTAAAGAATG -3' |

Gene accession number*:*
*OsAGPLI*: ADP-glucose pyrophosphorylase large subunit I (D50317)
*OsAGPLII*: ADP-glucose pyrophosphorylase large subunit II (U66041)
*OsAGPLIII*: ADP-glucose pyrophosphorylase large subunit III (AK069296)
*OsAGPSIIb*: ADP-glucose pyrophosphorylase small subunit IIb (XM_015756738)
*OsGBSSI:* granule-bound starch synthase I (X62134)
*OsSBEI*: Starch branching enzyme I (AF136268)
OsSSI: starch synthase I *(D16202)*
*OsSSIIa:* Starch synthase IIa *(AF419099)*
*OsSSIIb:* Starch synthase IIb *(AF395537)*
*OsSSIIc:* starch synthase IIc (AF383878)
*OsSSIIIa:* starch synthase IIIa (AY100469)
*OsSSIIIb:* Starch synthase IIIb *(AF432915)*

**Table S2** Comparison of apparent amylose content in endosperm among Wt, *OEtr-1* and *Ri-1*.

| Genotype | Amylose (%) |
|---|---|
| Wt (TNG67) | $23.35 \pm 0.27^{ab}$ |
| *OEtr-1* | $14.74 \pm 0.33^{c}$ |
| *Ri-1* | $26.15 \pm 0.48^{a}$ |

Mean values calculated from three independent experiments. All data are presented as mean ± SE, n=20. Values with different lower case letters are significantly different ($P < 0.05$). Statistical significant is determined by t-test.

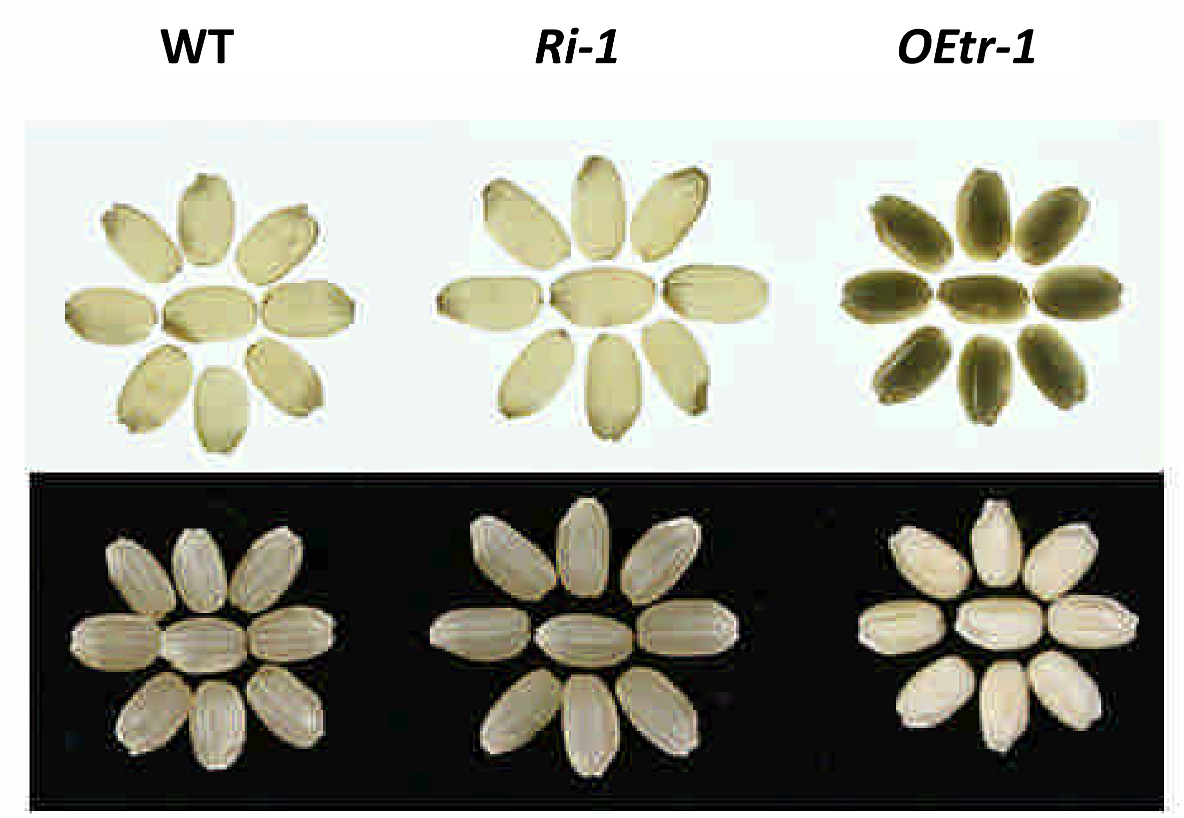


**Figure S1**. Comparison of the transparent appearance of endosperm between Wt, *OEtr-1* and *Ri-1* grains. Rice grains are illuminated with backlight (top panel) or normal lighting (bottom panel) conditions.

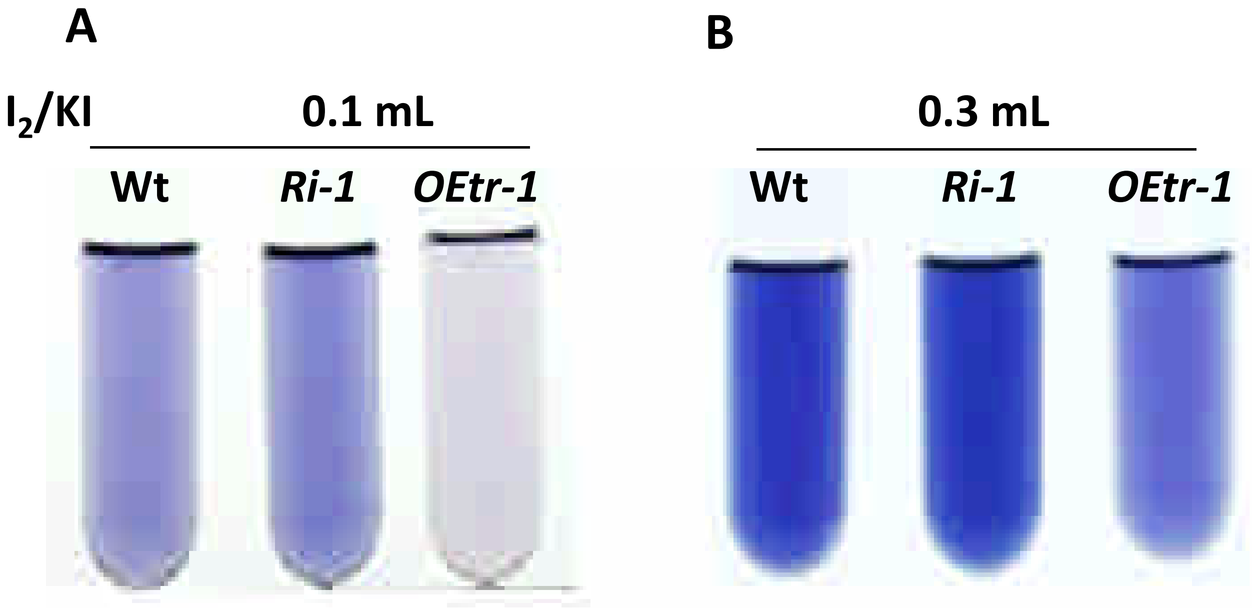


**Figure S2**. Iodine-staining of endosperm starch in Wt, *OEtr-1* and *Ri-1*. Twenty milligrams of endosperm powder were treated with 1N NaOH as described in materials and methods. Supernatants of the starch samples was mixed with (A) 0.1 mL or (B) 0.2 mL $I_2$/KI solution.

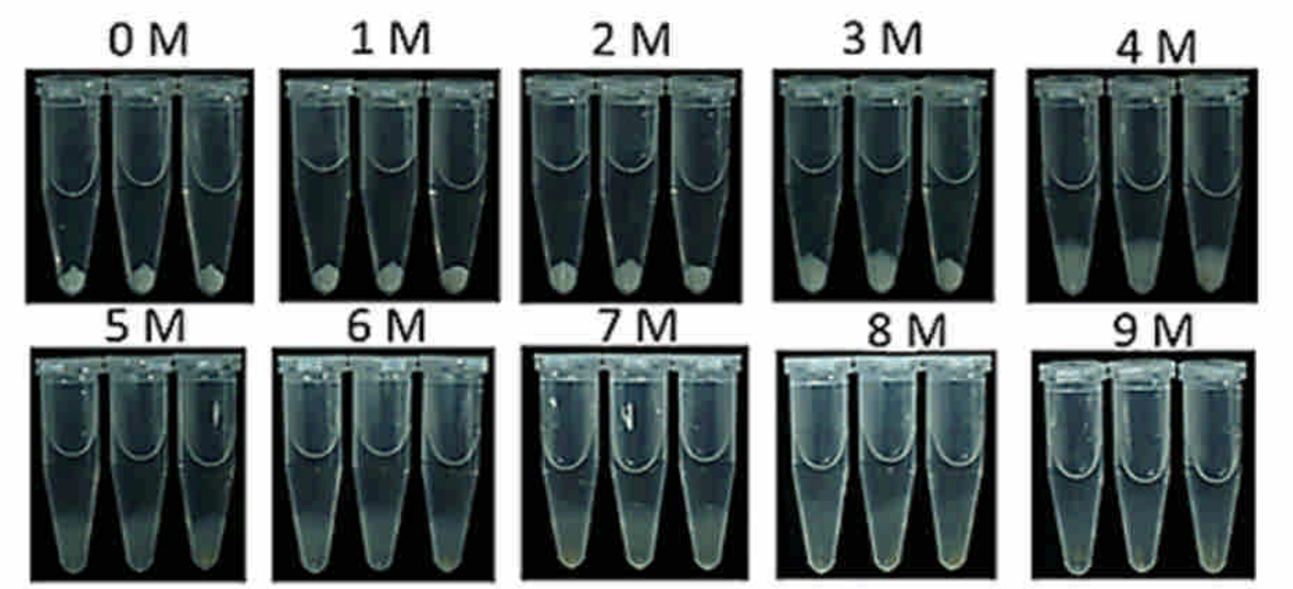

Wt *Ri-1 OEtr-1* Wt *Ri-1 OEtr-1* Wt *Ri-1 OEtr-1* Wt *Ri-1 OEtr-1* Wt *Ri-1 OEtr-1*

**Figure S3**. Effect of various concentration of urea on the gelatinization properties of endosperm starch in Wt, *OEtr-1* and *Ri-1*.
Twenty milligrams of rice powder in an eppendorf tube was mixed with 1 mL of urea solution and shaken for 24 h at 25°C.
After centrifugation, the volume of the gelatinization of starch swollen sediment was measured and recorded.

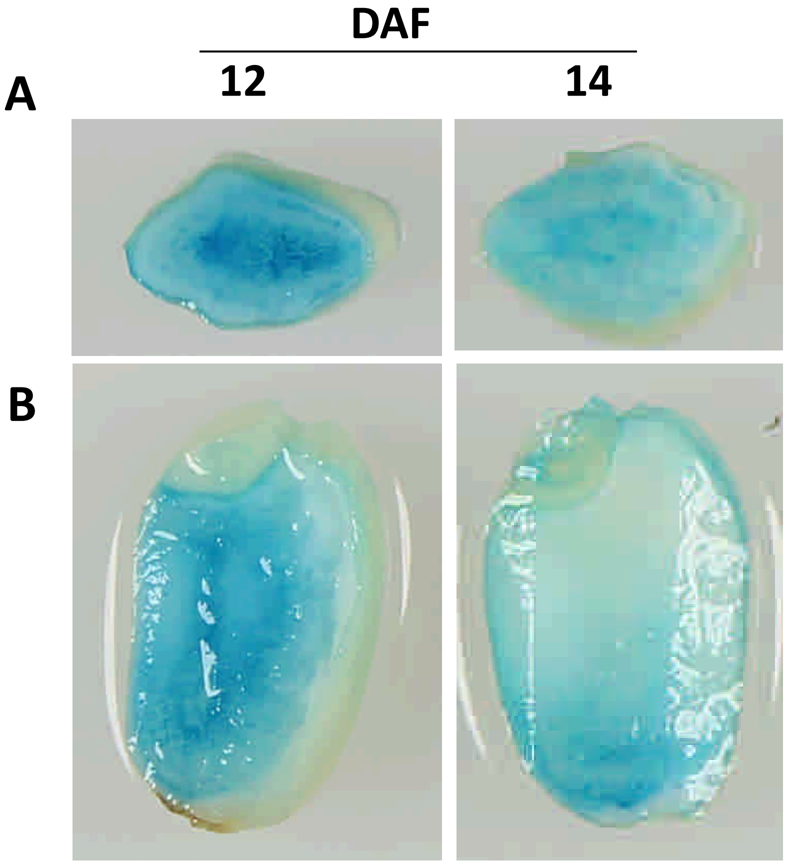


**Figure S4**. Histochemical GUS (β-glucuronidase) activity staining in cross and longitudinal section developing rice seeds. Immature rice seeds were collected at 12 and 14 DAF, respectively. The lemma and palea was completed removed carefully from the immature seeds. The cross section (A) and longitudinal section (B) of the seeds were cutted with a knife. The cutted of immature grains were stained and preserved in 70% ethanol and photographed. DAF: days after flowering.
